# A Toddler with Spontaneous Pneumomediastinum

**DOI:** 10.5811/cpcem.2017.5.33987

**Published:** 2017-10-03

**Authors:** Jessica L. Chow, Israel Green-Hopkins, Christopher R. Peabody

**Affiliations:** * University of California, San Francisco, Zuckerberg San Francisco General Hospital, Department of Emergency Medicine, San Francisco, California; † UCSF Benioff Children’s Hospital, Department of Emergency Medicine, Division of Pediatric Emergency Medicine, San Francisco, California

## CASE PRESENTATION

An 18-month-old female with unremarkable birth history presented to the emergency department (ED) for drooling and “difficulty breathing.” She had three days of cough and rhinorrhea, but otherwise no reports of fever, vomiting, trauma, foreign-body ingestion or aspiration. Her vaccinations were up to date.

**Image 1:**
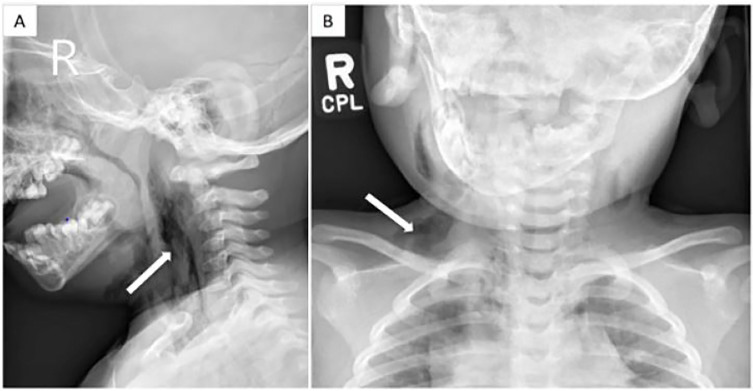
Neck (A) and chest (B) radiographs showing retropharyngeal and subcutaneous emphysema (arrows) tracking along bilateral facial and neck planes, predominantly the right carotid and supraclavicular region, and inferiorly into the mediastinum.

**Image 2:**
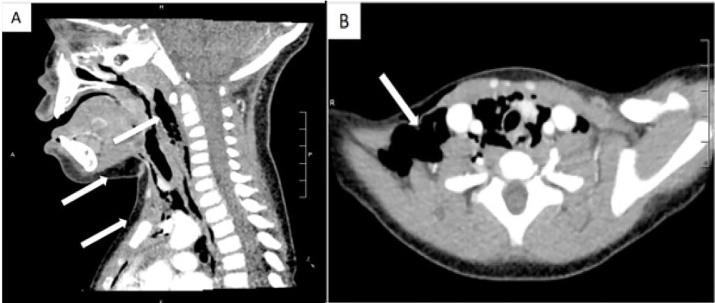
Computed tomography of the neck (A) and chest (B) confirmed subcutaneous air (arrows) tracking inferiorly into the mediastinum, without abscesses or necrotic nodes.

Her vital signs included blood pressure 143/98 mmHg, pulse 135 beats per minute, respiration 20 breaths per minute, temperature 36.ºC, oxygen saturation 98% on room air. Her examination was remarkable for pooled oral secretions and preferential rightwards neck tilt. She was without stridor or wheezing. She had no neck masses, tongue swelling, or crepitus. She was initially started on broad-spectrum antibiotics. A lateral neck and chest radiograph revealed retropharyngeal and subcutaneous emphysema tracking inferiorly into the mediastinum (Image 1). Bedside nasal endoscopy showed a patent airway and no masses. A computed tomography (CT) of the neck and chest (Image 2) and esophagram were otherwise unremarkable.

## DISCUSSION

Spontaneous pneumomediastinum (SPM) is an uncommon, often benign, condition in children, occurring in a bimodal distribution: six months–4 years and 15–18 years.^1^ Primary SPM occurs in the absence of underlying lung pathology, whereas secondary SPM occurs in the setting of underlying lung disease. One in five cases of SPM is associated with asthma.^1^–^2^ Common triggers include bronchospasm, respiratory tract infections (e.g., bronchopneumonia, bronchiolitis), and valsalva maneuvers.^1^ Life-threatening etiologies such as esophageal rupture, tension pneumothorax, and necrotizing mediastinitis are rare, but also should be queried on initial evaluation.

Young patients with SPM often present with acute chest pain or dyspnea;^2^ however, in pre-verbal children, it may be more difficult to localize symptoms. Subcutaneous emphysema is palpable in approximately 60% of patients.^1^ Chest radiographs diagnose 99.5% of SPM cases.^1^ Ultrasound detection of SPM has also been noted in case reports.^3^ Given a relatively benign clinical course, isolated SPM management in an otherwise well-appearing child includes a four-hour period of ED observation after diagnosis, treatment of associated disorders (e.g., asthma), and discharge home with a caregiver and close follow-up without hospitalization.^3^ If the assessment suggests primary SPM, advanced imaging such as esophagrams or CT is unnecesary.^4^ However, children who appear in distress or with potentially life-threatening causes of SPM may need further evaluation, including additional imaging or hospitalization. Most SPM resolve in 1–2 weeks with a <2% recurrence rate.^1^–^2^,^4^

**CPC-EM Capsule**

What do we already know about the clinical entity?

Spontaneous pnuemomediastinum (SPM) is an uncommon, often benign condition in children. Primary SPM may not warrant advanced imaging.

What is the major impact of the image?

The plain film image represents a diagnosis of benign spontaneous pneumomediastinum that emergency medicine physicians who care for pediatric patients may encounter.

How might this improve emergency medicine practice?

In a well-appearing child with isolated SPM and no life-threatening etiologies, management recommendations include an observation period without extensive work-up.

## References

[1] Gasser CR, Pellaton R, Rochat CP (2017). Pediatric spontaneous pneumomediastinum: narrative literature review. Pediatr Emerg Care.

[2] Bullaro FM, Bartoletti SC (2007). Spontaneous pneumomediastinum in children: a literature review. Pediatr Emerg Care.

[3] Ng L, Saul T, Lewiss RE (2013). Sonographic evidence of spontaneous pneumomediastinum. Am J Emerg Med.

[4] Fitzwater JW, Silva NN, Knight CG (2015). Management of spontaneous pneumomediastinum in children. J Pediatr Surg.

